# Analysis of the Stochastic Quarter-Five Spot Problem Using Polynomial Chaos

**DOI:** 10.3390/molecules25153370

**Published:** 2020-07-24

**Authors:** Hesham AbdelFattah, Amnah Al-Johani, Mohamed El-Beltagy

**Affiliations:** 1 Engineering Mathematics and Physics Department, Engineering Faculty, Cairo University, Giza 12613, Egypt; heshammustafa1992@gmail.com; 2 Mathematics Department, Science Faculty, Tabuk University, Tabuk 71491, Saudi Arabia; aalgohani@ut.edu.sa

**Keywords:** porous media, quarter-five spot problem, random variations, polynomial chaos, Karhunen-Loeve decomposition

## Abstract

Analysis of fluids in porous media is of great importance in many applications. There are many mathematical models that can be used in the analysis. More realistic models should account for the stochastic variations of the model parameters due to the nature of the porous material and/or the properties of the fluid. In this paper, the standard porous media problem with random permeability is considered. Both the deterministic and stochastic problems are analyzed using the finite volume technique. The solution statistics of the stochastic problem are computed using both Polynomial Chaos Expansion (PCE) and the Karhunen-Loeve (KL) decomposition with an exponential correlation function. The results of both techniques are compared with the Monte Carlo sampling to verify the efficiency. Results have shown that PCE with first order polynomials provides higher accuracy for lower (less than 20%) permeability variance. For higher permeability variance, using higher-order PCE considerably improves the accuracy of the solution. The PCE is also combined with KL decomposition and faster convergence is achieved. The KL-PCE combination should carefully choose the number of KL decomposition terms based on the correlation length of the random permeability. The suggested techniques are successfully applied to the quarter-five spot problem.

## 1. Introduction

Fluid flow in porous media is a subject of great interest due to its wide range of applications such as water/oil flow in underground reservoirs, modeling the underground spreading of toxic, and biological applications such as the transport in lungs and arteries [1]. When dealing with porous media, such as geological formations, we do not have complete information about their properties, such as permeability and porosity. This leads to uncertainty about the values of the formation properties and, in turn, in calculating flow in such formations [2]. For evaluating such uncertainties, a probabilistic framework is to be used. The input data are considered as random quantities, which are described in terms of stochastic processes.

Uncertainty quantification (UQ) methods are used for analyzing the system using a model by the parameterization of the uncertain input data (such as permeability) using a set of independent random variables. Then, the model is solved to determine the response of the system to the given random input data [3]. 

Most of the numerical models used for uncertainty quantification are based on the Monte Carlo (MC) sampling method [4,5,6,7,8,9]. This method is known for the simplicity of its implementation; however, it requires a large number of iterations of the numerical model in order to provide reliable results and therefore may be not feasible for complex simulators. In order to overcome these limitations, other variants to the standard MC method were developed such as the multilevel Monte Carlo method (MLMC) [4], Sequential Monte Carlo (SMC) Methods [5], and quasi-Monte Carlo methods [6]. In addition to the MC method and its variations, other approaches including analytical models [7] and reduced-order models [8,9] were introduced. The Karhunen-Loève (KL) expansion is an order reduction method that can be used to provide a low-dimensional representation for a Gaussian random field by performing a projection of the original system with respect to a set of spatially dependent basis and a set of independent standard normal random coefficients, where the eigenvalues and eigenvectors of the autocorrelation function of the Gaussian field are utilized in the expansion [8,9,10].

Another method is the polynomial chaos expansion (PCE) [11], where the random variables are expanded using polynomial chaos basis, then a set of equations are solved using the Galerkin technique to determine the expansion coefficients [12]. For the classical polynomial chaos expansion, the polynomial chaos basis is the Hermite polynomials, and the random variables are Gaussian. Theoretically, convergence is achieved for any $L_{2}$ functionals in the random space [13]; however, only Gaussian and near-Gaussian random fields achieve optimal convergence rate. A more general approach, the generalized polynomial chaos (gPC), was proposed in [14], where the random variables are not restricted to Gaussian random variables and the expansion is in terms of polynomials chosen from the hypergeometric polynomials of the Askey scheme [15]. However, gPC can be used only when the input pdf is known in order to choose the proper basis functions, which is not the case in many applications [16]. To overcome this issue, the arbitrary polynomial chaos (aPC) technique was proposed [16,17], where in aPC, the polynomial chaos expansion is based on the moments of the random data instead of the PDF.

When dealing with Gaussian processes, the convergence of the KL expansion is optimal [18], however, the convergence rate may be much slower for other types of processes where optimal convergence is achieved based on the particular choice, made a priori, of the basis functions used in the expansion. Therefore, PCE and KL become less accurate for nonlinear dynamics and long-time integrations due to the fixed basis and the increasing complexity with the time. To overcome that issue, the dynamically-orthogonal (DO) [19] and the bi-orthogonal (BO) [20] techniques were introduced to allow for time-dependent basis, and therefore, more accurate for long time intervals. In both techniques, the stochastic coefficients and the spatial basis evolve according to the stochastic partial differential equation (SPDE) while preserving an orthonormality conditions for the basis functions.

The purpose of this paper is to solve one of the standard porous media problems, namely, the quarter-five spot problem after imposing uncertainty to the problem. The quarter five sport problem is used to model the case when a regular set of injection and production wells is present in the reservoir; as this problem already illustrates the features and difficulties of the general problem [21]. The problem is solved using both PCE and KL techniques and the results are compared with the MC method to verify the efficiency of each technique.

The remainder of this paper is organized as follows. First, in Section 2, the mathematical model is introduced, then the details of the problem are given in Section 3. Finally, in Section 4, results and conclusions are discussed.

## 2. Mathematical Statement of the Quarter-Five Spot Problem

### 2.1. The Deterministic Problem

Consider the problem of fluid flow in porous medium for a single-phase flow with an incompressible rock and fluid under isothermal conditions [22].

The problem is presented by the following partial differential equation (PDE):

(1) $$\nabla .\left[\frac{-K}{\mu }\left(\nabla p+\rho g\nabla z\right)\right]=\frac{q}{\rho \;},$$

where ρ denotes the density of the fluid, q denotes sources and sinks (out flow and in flow of fluid per unit volume), K denotes the permeability, z is the vertical coordinate, and μ is the fluid viscosity.

Let G=−g∇z denote the downward force for gravity. Equation (1) can be expressed as

(2) $$\nabla .\left(\left(\frac{-K}{\mu }\frac{\partial P}{\partial x},\frac{-K}{\mu }\frac{\partial P}{\partial y}\right)+\left(-\rho \frac{\partial G}{\partial x},-\rho \frac{\partial G}{\partial y}\right)\right)=\frac{q}{\rho \;},$$

As gravity forces are approximately constant inside a reservoir domain, we get

(3) $$\frac{\partial G}{\partial x}=\frac{\partial G}{\partial y}=0,$$

Therefore,

(4) $$\nabla .\left(-K\frac{\partial P}{\partial x},-K\frac{\partial P}{\partial y}\right)=\frac{q}{\rho \;},$$

where we assumed constant viscosity μ.

If we assume water phase with ρ=1, we get Equation (1) in the form

(5) $$-K\frac{\partial^{2}P}{\partial x^{2}}-K\frac{\partial^{2}P}{\partial y^{2}}-\frac{\partial K}{\partial x}\frac{\partial P}{\partial x}-\frac{\partial K}{\partial y}\frac{\partial P}{\partial y}=q,$$

or in compact form,

(6) $$\nabla .\;\vec{v}=q,\;\vec{v}=-\left(K\nabla p\right),$$

where $\vec{v}$ is the macroscopic Darcy velocity.

Equation (6) is a second order linear PDE. Additionally, Equation (6) is elliptic as the eigenvalues of its coefficient matrix are all negative.

### 2.2. The Stochastic Problem

Due to our incomplete knowledge about the medium properties, subsurface flow involves some degree of uncertainty, and therefore we need a stochastic description of the medium properties [23]. We can impose uncertainty to the quarter-five spot problem by considering the hydraulic conductivity (permeability) as a random field K=K(x, ω), where x  is the spatial coordinate and ω is the random outcome on a given probability space Ω. This, in turn, makes the solution P a random field. In our model, the boundary conditions and the source term are kept deterministic.

Thus, Equation (6) becomes an SPDE stated as

(7) ∇· (−K(x;ω)∇p(x;ω))=q,

The PCE method can be used to represent a second order process $f\in L_{2}\left(\Omega \right)$, where $L_{2}\left(\Omega \right)$ is the space of square integrable $L_{2}\;$ functions, such that a random field u(x;ω) can be expressed as [24]

$$\;u\left(x;\omega \right)=u_{0}\left(x\right)+\overset{\infty }{\underset{i_{1}=1}{\sum }}u_{i_{1}}\left(x\right)\varphi_{1}\left(\xi_{i_{1}}\left(\omega \right)\right)\;$$

$$+\overset{\infty }{\underset{i_{1}=1}{\sum }}\overset{i_{1}}{\underset{i_{2}=1}{\sum }}u_{i_{1}i_{2}}\left(x\right)\varphi_{2}\left(\xi_{i_{1}}\left(\omega \right),\xi_{i_{2}}\left(\omega \right)\right)$$

(8) $$+\overset{\infty }{\underset{i_{1}=1}{\sum }}\overset{i_{1}}{\underset{i_{2}=1}{\sum }}\overset{i_{2}}{\underset{i_{3}=1}{\sum }}u_{i_{1}i_{2}}\left(x\right)\varphi_{2}\left(\xi_{i_{1}}\left(\omega \right),\xi_{i_{2}}\left(\omega \right),\xi_{i_{3}}\left(\omega \right)\right)+\ldots ,$$

To simplify writing the equations, we can eliminate the arguments when there is no ambiguity. $\varphi_{n}\left(\xi_{i_{1}},\xi_{i_{2}},\ldots ,\xi_{i_{n}}\right)$ denotes the orthogonal polynomial of degree n with respect to the multidimensional random variables $\xi =\left(\xi_{i_{1}},\;\ldots ,\xi_{i_{n}},\ldots \right)$.

If $\xi_{i_{1}},\xi_{i_{2}},\ldots ,\xi_{i_{n}}$ are independent standard Gaussian random variables, then $\varphi_{n}\left(\xi_{i_{1}},\xi_{i_{2}},\ldots ,\xi_{i_{n}}\right)$ represent the multidimensional Hermite Polynomials of degree n expressed as

(9) $$\varphi_{n}\left(\xi_{i_{1}},\ldots ,\xi_{i_{n}}\right)={\left(-1\right)}^{n}e^{\frac{1}{2}\xi^{T}\xi }\frac{\partial^{n}}{\partial \xi_{i_{1}}\ldots \partial \xi_{i_{n}}}\left[e^{\frac{1}{2}\xi^{T}\xi }\right]$$

For Gaussian random variables, Hermite polynomials are the most suitable orthogonal basis [11]. For other distributions, the basis polynomials are selected to optimize the convergence rate. This generalization is commonly known as gPC [25].

For computational purposes, if we have a random vector ξ of dimension N, then Equation (8) can be written in terms of a finite number of Hermite polynomials as

(10) $$u\left(x;\omega \right)=\overset{P}{\underset{i=0}{\sum }}u_{i}\left(x\right)\psi_{i}\left(\xi \right),$$

where the total number of expansion terms P+1 is determined by the random vector dimension N and the order d of the PCE [23].

(11) $$P+1=\frac{\left(N+d\right)!}{N!d!},$$

The functions $\psi_{i\;}$ construct orthogonal basis in $L_{2}\left(\Omega \right)$ with the orthogonality relation [24]

(12) $$\langle \psi_{i},\psi_{j}\rangle =\langle \psi_{i}{}^{2}\rangle \delta_{ij},$$

where $\delta_{ij}$ is the Kronecker delta and $\langle .,.\rangle$ is the ensemble average, that is, the inner product in the Hilbert space of the variables ξ defined by

(13) $$\langle f\left(\xi \right),g\left(\xi \right)\rangle =\underset{\Omega }{\int }f\left(\xi \right)g\left(\xi \right)W\left(\xi \right)d\xi ,$$

where W(ξ) is the weighting function corresponding to the polynomial chaos basis $\psi_{i}$.

In case that the polynomial chaos basis are the Hermite polynomials, W(ξ) is given by

(14) $$W\left(\xi \right)=\frac{1}{\sqrt{{\left(2\pi \right)}^{N}}}e^{\frac{1}{2}\xi^{T}\xi },$$

where N is the number of independent Gaussian random variables.

The mean square error of the truncated PCE expansion, u(x;ω), is expressed as

(15) $$\;e\left(x\right)=\sqrt{E{\left[u\left(x;\omega \right)-u_{e}\left(x;\omega \right)\right]}^{2}},$$

where $u_{e}\left(x,\;\omega \right)$ is the exact solution and E is the expectation operator.

For the case of univariate Gaussian stochastic germ, the following theorem provides an error bound.

Theorem 1 [25]. Given $u_{e}\left(x,\;\omega \right)\in L_{2}$, being k times continuously differentiable, the convergence rate

(16) $$\|u_{e}\left(x,\;\omega \right)-\overset{P}{\underset{i=0}{\sum }}u_{i}\left(x\right)W_{i}\left(\xi \right)\|\;\leq \frac{1}{\prod_{i=0}^{k-1}\sqrt{\left(n-i+1\right)}}\left\|u^{\left(k\right)}\right\|,$$

is obtained for an approximation of $u_{e}\left(x,\;\omega \right)$ by a finite number of Hermite polynomials ${\left\{W_{i}\right\}}_{i=0}^{P},$ where the norm ‖.‖ is induced by the inner product (14).

The KL expansion can be viewed as a special case of the PCE where only first-order polynomials have non-zero coefficients [26]. The KL expansion involves the spectral expansion of the correlation function of the process under consideration and is used to reduce the dimensionality of the random space [24]. Consider a second-order process Z(x;ω) with a correlation function C(x,y). The correlation function should be real, symmetric, and positive definite [14].

We can represent Z(x;ω) as

(17) $$Z\left(x;\omega \right)=E\left[Z\left(x,.\right)\right]+\overset{\infty }{\underset{i=1}{\sum }}\sqrt{\lambda_{i}}\phi_{i}\left(x\right)\;\xi_{i}\left(\omega \right),$$

where Z(x,.):Ω→R is a random variable, E[Z(x,.)] is mean of the process, $\xi_{i}\left(\omega \right)$ a set of uncorrelated random variables, $\lambda_{i}$ and $\phi_{i}\left(x\right)$ are the eigenvalues and eigenfunctions of the correlation function, respectively, i.e.,

(18) $$\underset{\Omega }{\int }C\left(x,y\right)\phi_{i}\left(y\right)dy=\lambda_{i}\phi_{i}\left(x\right),$$

The KL expansion is effective when the correlation function is known, as it is characterized by an optimal convergence in the mean square sense [25]. However, the correlation function might not be known in some cases, which is a limitation of the KL expansion method.

## 3. Solution Implementation

### 3.1. The Deterministic Problem

Recall Equation (6):

(19) $$\nabla .\vec{v}=q,\;\vec{v}=-\left(K\nabla p\right),$$

To have a complete solution, we need to specify the boundary conditions. Closed flow systems where there is no fluid flow across the external boundaries are modeled by specifying homogeneous Neumann boundary conditions:

(20) $$\vec{v.}\vec{n}=0,\;for\;\vec{x}\in \partial \Omega ,$$

where $\vec{n}$ is the normal at the boundary ∂Ω of the computational domain and $\vec{x}$ is any spatial point that exists on the domain’s boundary  ∂Ω.

There are several numerical methods used to solve (4), three of these methods were discussed in [22]. The first method is a cell-centered finite-volume method called the two-point flux-approximation (TPFA) scheme that is widely used in the oil industry. The TPFA scheme uses two cells pressure averages to approximate the flux $v_{i,k}$ across each face of the cell. In some cases, the orientation of the computational grid will affects the results of the TPFA scheme; thus, another finite-volume method called the multi-point flux-approximation (MPFA) scheme is used to overcome the inconsistencies of the TPFA scheme. Instead of using only two points such as the TPFA, the MPFA scheme utilizes each of the neighboring cell averages to approximate the flux. Another alternative to the MPFA scheme is the mixed finite-element method where the fluxes over cell edges are considered as unknowns in addition to the pressure values. However, due to the simplicity and computational speed of the TPFA, we will restrict our attention to this method. First, we write Equation (17) in the integral form inside a cell $\Omega_{i}$ as the control volume.

(21) $$\underset{\partial \Omega_{i}\;}{\int }\vec{v}.\vec{n}\mathrm{ds}=\underset{\Omega_{i}\;}{\int }q\mathrm{dV},$$

To compute the flux $v_{i,k}$ across each face of the cell we use Darcy’s law,

(22) $$v_{i,k}=\int_{\Gamma_{i,k}\;}\vec{v}.\vec{n}\mathrm{ds},\;\Gamma_{i,k}=\partial \Omega_{i}\cap^{\Omega }\partial \Omega_{k},$$

where $\Gamma_{i,k}$ Is the interface between the two cells’ boundaries $\partial \Omega_{i}\;$ and$\;\partial \Omega_{k}$ with an area$\;A_{i,k}$ and a normal vector $\;\vec{n_{i,k}}$, $\Gamma_{i,k}$ is the opposite interface and has identical area$\;A_{i,k}=A_{k,i}$ but opposite normal vector$\;\vec{n_{i,k}}=-\vec{n_{k,i}}$.

The integral can be approximated as

(23) $$v_{i,k}\approx -A_{i,k}\left(K_{i}\nabla p_{i,k}\right).\vec{n_{i,k}},$$

where $\nabla p_{i,k\;}$ is the pressure gradient at the centroid of the interface $\;\Gamma_{i,k}.$ Finite difference is used to approximate $\nabla p_{i,k}$ using the pressure $\tilde{p_{i,k}}$ at the interface’s centroid and the average pressure $p_{i}\;$ inside the cell${\;\Omega }_{i}$,

(24) $$v_{i,k}\approx -A_{i,k}K_{i}\frac{\left(p_{i}-\tilde{p_{i,k}}\right)\vec{x_{i,k}}}{{\vec{\left\|x_{i,k}\right\|}}^{2}}.\vec{n_{i,k}}=T_{i,k}\left(p_{i}-\tilde{p_{i,k}}\right),$$

where $\vec{x_{i,k}}\;$ is the position vector between the center of the cell ${\;\Omega }_{i}$ and the centroid of the interface $\;\Gamma_{i,k}$, and $T_{i,k}$ is the one-sided transmissibility associated with the cell ${\;\Omega }_{i}.$ Therefore, we have for cell ${\;\Omega }_{i}$ the relation

(25) $$T_{i,k}{}^{-1}v_{i,k}=\left(p_{i}-\tilde{p_{i,k}}\right),$$

Similarly, we have for cell${\;\Omega }_{k}$ the relation

(26) $$T_{k,i}{}^{-1}v_{k,i}=\left(p_{k}-\tilde{p_{k,i}}\right),$$

By imposing the continuity of fluxes, we have $v_{i,k}=-v_{k,i}=v_{ik}$ and $\;\tilde{p_{i,k}}=\tilde{p_{k,i}}=\tilde{p_{ik}}$, and after eliminating $\;\tilde{p_{ik}}$ we get

(27) $$\;v_{ik}={\left[T_{i,k}{}^{-1}+T_{k,i}{}^{-1}\right]}^{-1}\left(p_{i}-{\;p}_{k}\right)=T_{ik}\left(p_{i}-{\;p}_{k}\right),$$

where $T_{ik}$ is the transmissibility associated with the connection between the two cells $\Omega_{i}\;$ and $\;\Omega_{k}$. Therefore, TPFA uses the cell averages $p_{i}$ and $p_{k}$, to approximate the flux across the interface $\Gamma_{i,k}$ between the cells$\;\Omega_{i}\;$ and $\;\Omega_{k}$.

Finally, when substituting $v_{ik}$ in Equation (19) we get the following system of equations,

(28) $$\sum_{k}T_{ik}\left(p_{i}-{\;p}_{k}\right)=q_{i},$$

where

(29) $$T_{ik}=2\Delta y{\left[\frac{\Delta x_{i}}{K_{i}}+\frac{\Delta x_{k}}{K_{k}}\right]}^{-1},$$

Therefore we get the following system of equations,

(30) Au=f,

where A is a sparse, penta-diagonal symmetric matrix. Moreover, when dealing with a homogenous permeability field, A is a Toeplitz matrix. The system is made positive definite, which can be effectively solved using the Cholesky decomposition algorithm [27].

#### Numerical Example

**Figure 1:**
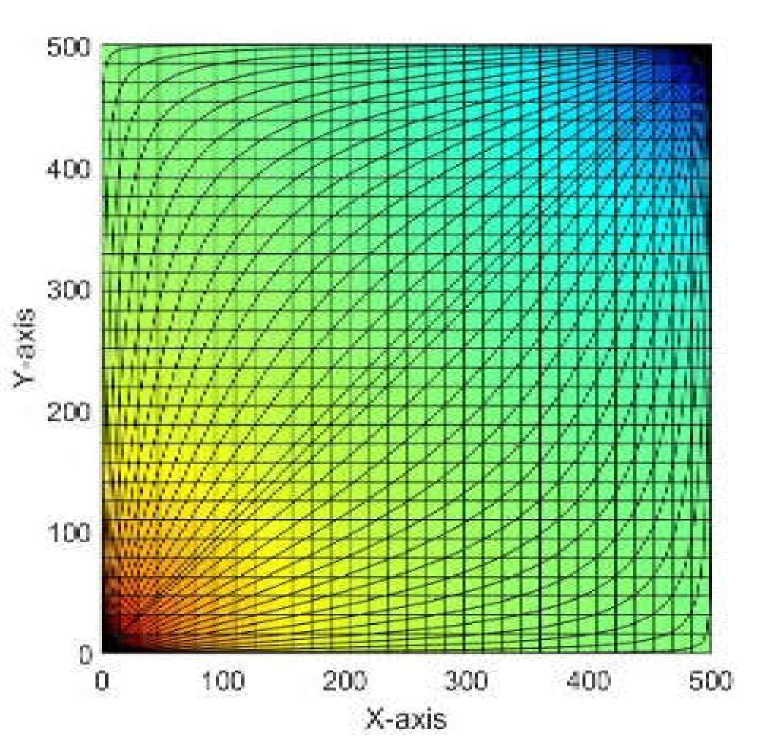
Solution of the quarter-five spot problem on a 32×32  uniform grid. The plot shows the pressure distribution on each point in the grid, where the highest pressure values are in the bottom left corner and the pressure decreases gradually until it reaches the lowest value at the top right corner.

Equation (4) is solved on a 32×32  uniform Cartesian grid covering a $500\times 500\;m^{2}$ area with a homogeneous isotropic permeability K=100 md with porosity of 0.2  for all points in the grid. There is an injection well at the origin with a flow rate of 1 $m^{3}/day$ and 4 production wells with flow rate of −1 $m^{3}/day$ at the points (±1, ±1). We use the homogeneous Neumann boundary conditions. The flow in the five-spot is symmetric about both the coordinate axes. Therefore, the domain can be reduced to a quarter of its size. This problem is called the quarter-five spot problem. Figure 1 shows the pressure distribution on the grid while illustrating the position of the injection and production wells on the bottom left and top right corners of the grid, respectively. The area of largest pressure’s value is the immediate zone surrounding the injection well (shown in red), then the pressure decreases gradually till it reaches its lowest value at the production well. In order to obtain a better view of the flow field, Pollock’s method [28] for semi-analytical tracing of streamlines is used to track the path of flow of the particles as they move from cell to cell through the cells’ centers. Additionally, traces are used, which are particles that flow with the fluid without changing the characteristics of the fluid.

The concentration of a tracer is given by a continuity equation

(31) $$\frac{\partial \left(\phi C\right)}{\partial t}+\nabla .\left(\vec{v}C\right)=q_{C},$$

where C is the concentration, $q_{C}$ is the inflow rate per unit volume for the tracer, and ϕ is the rock porosity. In the case of steady state flow, with tracers whose concentration do not change with fluid compression or expansion and with concentration equal to one at the inflow boundary, we get the simplified equation

(32) $$\nabla .\left(\vec{v}C\right)=q_{C}\;C_{inflow}=1,$$

We define the time-of-flight τ as the time required by a fluid particle to travel a given distance along a streamline. τ is given by the differential equation

(33) $$\vec{v}.\nabla \tau =\phi .$$

**Figure 2:**
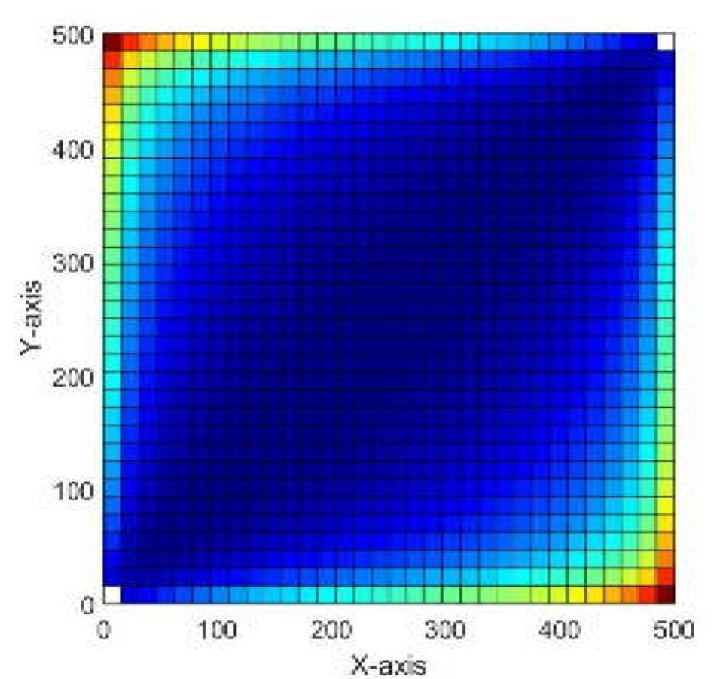
Total travel time to illustrate the areas of high speed flow and those of stagnant flow.

Applying the time flight to a tracer, the communication patterns of the reservoir can be studied effectively. Therefore, in order to estimate the speed of flow inside the reservoir, we solve equation (6) subject to τ=0 at the inflow to get the forward time-of-flight from inflow point and into the reservoir, and backward time-of-flight from outflow points and backwards into the reservoir. The sum of forward and backward times gives the total time required by a particle to enter the reservoir from inflow and travel through until it exits through the outflow. Figure 2 shows total travel time to illustrate the areas of high speed flow and those of stagnant flow.

### 3.2. The Stochastic Quarter-Five Spot Problem

We now proceed to solve Equation (7) while considering the uncertainty of the permeability field K(x;ω). The problem is first solved using only PCE, and then we will use KL expansion to reduce the dimension of the input space then apply PCE. For both scenarios, the pressure solution statistics are presented and the results are compared with the MC solution with a sample size of 10,000.

- First: Using PCE

Let permeability tensor K be a random field with a Gaussian distribution of mean 100 md and variance 0.5 md. Consequently, the pressure vector p and velocity vector $\vec{v}$ become stochastic processes. In order to apply PCE, we express K as a series of orthogonal basis functions $\psi_{i}$ (Hermite polynomials) and expansion coefficients $k_{i}$

(34) $$K\left(x;\omega \right)=\sum_{i=0}^{P}k_{i}\left(x\right)\psi_{i}\left(\xi \right)\;,$$

Accordingly,

(35) $$p\left(x;\omega \right)=\sum_{i=0}^{P}p_{i}\left(x\right)\psi_{i}\left(\xi \right)\;,$$

and

(36) $$q\left(x;\omega \right)=\sum_{i=0}^{P}q_{i}\left(x\right)\psi_{i}\left(\xi \right)\;,$$

substituting into Equation (7) and eliminating the arguments for brevity, we get

(37) $$\nabla .\left[-\sum_{i=0}^{P}k_{i}\psi_{i}\nabla \left(\sum_{j=0}^{P}p_{j}\psi_{j}\;\right)\right]=\sum_{i=0}^{P}q_{I}\psi_{I},$$

Which can be simplified to become

(38) $$\overset{P}{\underset{i=0}{\sum }}\overset{P}{\underset{j=0}{\sum }}-\left[k_{i}\nabla^{2}p_{j}+\nabla k_{i}.\nabla p_{j}\right]\psi_{i}\psi_{j}=\overset{P}{\underset{i=0}{\sum }}q_{i}\psi_{j}.$$

Applying inner product $\langle .,.\rangle$ with each polynomial $\psi_{k},\;k=0,\ldots ,P,$ and utilizing the orthogonality relation to get a set of P+1  deterministic equations of the form

(39) $$\sum_{i=0}^{P}\sum_{j=0}^{P}-\left[k_{i}\nabla^{2}p_{j}+\nabla k_{i}.\nabla p_{j}\right]e_{ijk}=q_{K}\langle \psi_{k}{}^{2}\rangle ,$$

where $e_{ijk}=\langle \psi_{i}\psi_{j}\psi_{k}\rangle ,$ which can be computed and stored priori [29]. We can apply the TPFA numerical scheme for this set of deterministic equations and obtain the pressure components $p_{i}\left(x\right)$.

- Second: Using KL expansion

As the realizations of a Gaussian distribution may take negative values, it is more practical to assume a log normal distribution for K. For that purpose, first we define a Gaussian process Z(x;ω)  with the KL expansion,

(40) $$Z\left(x;\omega \right)=E\left[Z\left(x,.\right)\right]+\sum_{i=1}^{M}\sqrt{\lambda_{i}}\phi_{i}\;\xi_{i},$$

where E[Z(x,.)] is the mean, and $\lambda_{i}$ and $\phi_{i}\left(x\right)$ are the eigenvalues and eigenfunctions of the exponential correlation function C(x,y), respectively. The correlation C(x,y) is of the form [30,31]

(41) $$C\left(x,y\right)=\sigma^{2}{}_{z}\exp\left(\frac{-\|x-y\|}{l}\right),x,y\;\epsilon D,$$

where l denotes the spatial correlation length and $\sigma^{2}{}_{z}\;$ is the process variance.

The permeability  K and  Z are related such that K is the exponential of Z and the PCE of K would be

(42) $$K\left(x;\omega \right)=e^{Z\left(x;\omega \right)}=\overset{P}{\underset{i=0}{\sum }}k_{i}\left(x\right)\psi_{i}\left(\xi \right)\;$$

To obtain the coefficients $k_{i}\left(x\right)$, we use the formula

(43) $$k_{i}\left(x\right)=\frac{\langle \psi_{i}\left(\eta \right)\rangle }{\langle \psi_{i}^{2}\rangle }\exp\left[E\left[Z\left(x,.\right)\right]+\frac{\sigma^{2}{}_{z}}{2}\right],\;\eta_{j}=\xi_{j}-z_{j}\;,$$

with$\;\psi_{i}\left(\eta \right)$ given in Table 1.

After computing the coefficients $k_{i}\left(x\right)$, we apply PCE to we get the following set of deterministic equations to be solved to get the pressure components using the TPFA scheme,

(44) $$\overset{P}{\underset{i=0}{\sum }}\overset{P}{\underset{j=0}{\sum }}-\left[k_{i}\nabla^{2}p_{j}+\nabla k_{i}.\nabla p_{j}\right]e_{ijk}\;=q_{K}\psi_{k}{}^{2},$$

where $e_{ijk}=\langle \psi_{i}\psi_{j}\psi_{k}\rangle$.

## 4. Results

### 4.1. Case of PCE Only

**Figure 3:**
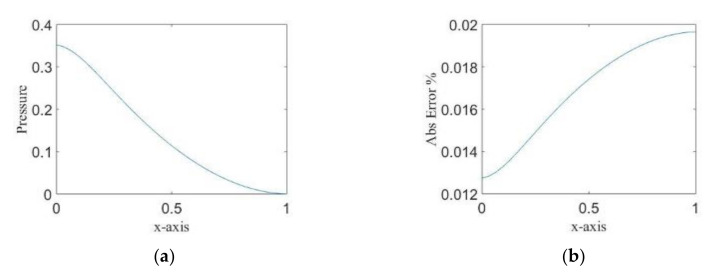
(**a**) The pressure mean solution using MC method. (**b**) The percentage of difference of solutions between MC and PCE methods.

We compare the differences in solutions obtained using MC and PCE methods. Figure 3 represents the normalized pressure mean solution produced by MC method (left) and the absolute error between the MC mean solution and the PCE mean solution (right). There was no considerable error between the two solutions (less than 0.02%) when assuming PCE with order d=2, which proves the efficiency of the method for linear problems even when taking a low order expansion.

**Figure 4:**
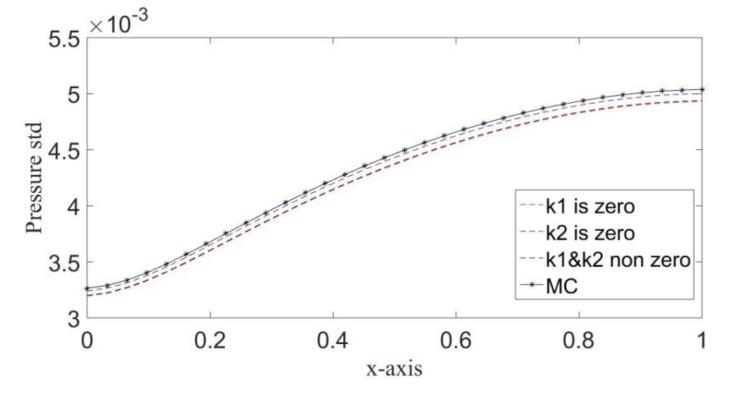
Comparing the pressure standard deviation resulting from MC and PCE methods.

Figure 4 shows variances in pressure solutions of MC and PCE methods when eliminating one of the PCE coefficients of the input random field K. It has been observed that the elimination has an insignificant impact on the accuracy of the solution. However, using more PCE coefficients tends to minimize the error between MC and PCE solutions. These results illustrate the fast convergence of the PCE solution for PDE (8), as assuming a higher-order PCE had a negligible effect on the accuracy of the solution.

Table 2 illustrates the percentage of the difference between MC and PCE solutions when eliminating the first PCE coefficient, i.e., $k_{1}=0,$ then the second PCE coefficient $k_{2}=0$, then without elimination. The minimum percentage of error results when using all PCE coefficients, while using only the second PCE coefficient. $k_{1}=0\;$ resulted in more accurate results than when $k_{2}=0$.

### 4.2. Case of KL with PCE

In the second case, we apply the KL expansion to the input, in order to reduce the dimension of the input space and then use PCE. The permeability field K is of log-normal distribution in this case. The MC method used for validation is modified by utilizing the KL expansion on a Gaussian field Z and constructing K by taking the exponential of Z in every iteration.

**Figure 5:**
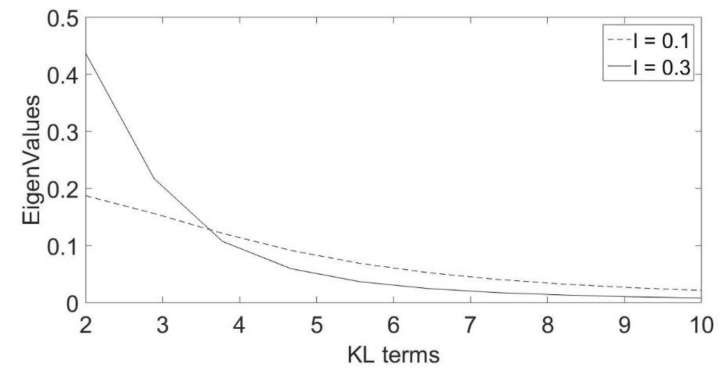
Comparing Eigenvalues convergence rate for l=0.1  and l=0.3.

First, we compare the convergence of the eigenvalues of the correlation function C(x,y) for different spatial correlation lengths. Figure 5 shows that as the correlation length decreases, the convergence would require more expansion terms. This can be further illustrated in Table 3, where the minimum number of KL terms required for convergence is provided for four different correlation lengths. We compared the solutions resulting from using PCE only and those resulting from using KL then PCE (KL-PCE) using different correlation lengths, where the results are compared against the MC solution. It has been noticed that there are no considerable differences in the mean solutions for all methods. However, the variance solution differs considerably based on the used method and the parameters $\sigma^{2}$ and l of the correlation function C(x,y).

The problem is solved using PCE of order d=1, and PCE of order d=2 after applying KL. The results show that PCE of order d=1 provides accurate results for low variance $\sigma^{2}$ values (less than 0.5). Even for highly heterogeneous mediums where we can set 

[32], PCE with first-order polynomials provides acceptable accuracy (less than 1% error when compared to MC) with no much need for higher-order PCE. However, for $\sigma^{2}>1$, using higher-order PCE is recommended for more accurate results. Therefore, we can apply KL in our problem before applying PCE as this leads to a faster convergence. It should be noted that when using KL the correlation length must be taken into consideration, as using higher correlation length would significantly reduce the accuracy of the solution if the minimum number of KL terms required for the eigenvalues convergence is not used. This can be observed in Table 4, where we compare the mean square error (MSE) percentages between MC and KL/PCE variance solutions.

## 5. Conclusions

In this paper, we studied the quarter-five spot problem. We solved both the deterministic problem and the stochastic problem after imposing uncertainty to the problem. We considered two cases for the stochastic problem: The first case is solved using both MC and PCE methods and the results are compared. It was shown that for the given problem, assuming a PCE with order d=2 produces an error in the mean solution less than 0.02%, while reducing the computational time to at least 30% of the time needed by the MC method. In the second case, we compared the effect of applying the KL expansion with different correlation function parameters before applying the PCE. It has been shown that for systems with low heterogeneity (low values of process variance $\sigma^{2}$), PCE with first-order polynomials provides accurate results (less than 1% error). Even for a highly heterogeneous medium with $\sigma^{2}=1,$ PCE with first-order polynomials provides acceptable accuracy in most cases. However, if the value of $\sigma^{2}$ increases, higher-order PCE may be required, and the KL expansion can be applied for better convergence of the PCE. When using the KL expansion, the correlation length of the correlation function should be taken into consideration before choosing the number of KL expansion terms. It was observed that a correlation function with a higher correlation length may require using the full number of KL expansion terms needed for the convergence of the eigenvalues of the correlation function. This study can be extended to predict the expected behavior of the general case when there is a system of a reservoir with injection/production wells with uncertain parameters and/or boundary conditions.

## Figures and Tables

**Table 1:** $\;\psi_{i}\left(\eta \right)$ used to evaluate the PC coefficients $k_{i}\left(x\right)$ [11].

$\psi_{i}\left(\xi \right)$	$\psi_{i}\left(\eta \right)$	$\psi_{i}\left(\eta \right)$
$\xi_{i}$	$\eta_{i}+z_{i}$	$z_{i}$
$\xi_{i}\xi_{j}-\delta_{ij}$	$\left(\eta_{i}+z_{i}\right)\left(\eta_{j}+z_{j}\right)\_-\delta_{ij}$	$z_{i}z_{j}$
$\xi_{i}\xi_{j}\xi_{k}-\xi_{i}\delta_{jk}-\xi_{j}\delta_{ik}-\xi_{k}\delta_{ij}$	$\left(\eta_{i}+z_{i}\right)\left(\eta_{j}+z_{j}\right)\left(\eta_{j}+z_{j}\right)\_-z_{i}\delta_{jk}-z_{j}\delta_{ik}-z_{k}\delta_{ij}$	$z_{i}z_{j}z_{k}$

**Table 2:** PCE vs. MC solution statistics.

	$k_{1}=0$	$k_{2}=0$	$k_{1}\&k_{2}\;non\;zero$
Mean difference percentage	0.0035	0.0039	0.0031
Variance difference percentage	2.56	2.61	2.52

**Table 3:** PCE vs. MC solution statistics.

Correlation Length l	l=0.1	l=0.3	l=0.5	l=1
Number of terms for convergence	>12	12	8	5

**Table 4:** PCE vs MC variances MSE.

Method	MSE% at σ=0.1	MSE% at σ=0.5	MSE% at σ=1
PCE	0.02	0.4	1
KL-PCE l=0.1	0.01	0.5	0.1
KL-PCE l=0.3	0.02	0.3	0.9
KL-PCE l=0.5	0.1	0.8	4
